# Polygenic scores and baseline cognitive function in midlife

**DOI:** 10.1002/alz70861_108618

**Published:** 2025-12-23

**Authors:** Nadia Dehghani, Rory Boyle, Dawei Xie, Xi Zuo, Shana D. Stites, Rebecca T. Brown, Corey T. McMillan

**Affiliations:** ^1^ Department of Neurology, University of Pennsylvania Perelman School of Medicine, Philadelphia, PA USA; ^2^ Department of Biostatistics, Epidemiology, and Informatics, University of Pennsylvania, Philadelphia, PA USA; ^3^ Department of Psychiatry, University of Pennsylvania Perelman School of Medicine, Philadelphia, PA USA; ^4^ Leonard Davis Institute of Health Economics, University of Pennsylvania, Philadelphia, PA USA; ^5^ Division of Geriatric Medicine, University of Pennsylvania Perelman School of Medicine, Philadelphia, PA USA

## Abstract

**Background:**

Midlife is a key life‐course period for understanding risk factors of cognitive decline. Despite growing evidence demonstrating polygenic contributions to age‐related disease risk, less is understood about how polygenic scores (PGS) may relate to cognitive traits in middle age. In a cross‐sectional analysis, we investigated how PGS for Alzheimer’s disease (AD), longevity, subjective‐wellbeing (based on life satisfaction and positive affect), and cognitive‐function may relate to cognition in a cohort of middle‐aged adults with normal cognition.

**Methods:**

The Health and Retirement Study (HRS) is an ongoing survey in Americans aged 50 years or older. We studied a cohort of 6615 individuals from the HRS (5359 European‐ancestry, 1256 African‐ancestry) aged 50‐56 years with normal cognition at baseline, as determined by the HRS cognitive test battery. PGS derived from genetic variants associated with traits through genome‐wide association studies in independent cohorts were available for all individuals. Linear regressions adjusted for baseline age, gender, and years of education were performed separately by genetically‐defined ancestral group.

**Results:**

Higher baseline cognition was observed in females (European‐ancestry β=0.72±0.08,*p*<0.001; African‐ancestry β=0.46±0.18,*p*=0.009) and individuals with more years of education (European‐ancestry β=0.39±0.02,*p*<0.001; African‐ancestry β=0.29±0.04,*p*<0.001). Higher cognitive‐function PGS related to higher cognition (European‐ancestry β=0.19±0.04,*p*<0.001; African‐ancestry β=0.20±0.09,*p*=0.024). In the European‐ancestry group, the cognitive‐function PGS partially mediated the relationship between education and cognition (proportion mediated in European‐ancestry=0.019, CI=0.010–0.030,*p*<0.001; proportion mediated in African‐ancestry=0.016, CI=‐0.002–0.050,*p*=0.072). The cognitive‐function PGS still partially mediated the relationship between education and cognition in a subset of the European‐ancestry group matched to the African‐ancestry group based on age, gender, education, and cognition (*n* =1256 European‐ancestry, proportion mediated=0.016, CI=0.002–0.040,*p*=0.016). Therefore, the lack of mediation in the African‐ancestry group is likely not due to smaller sample size. We did not observe associations between cognition and PGS for AD, longevity, and subjective‐wellbeing in either midlife ancestral group.

**Conclusions:**

In this HRS cohort, more years of education were related to higher baseline cognition, and in individuals with European‐ancestry, this relationship was partially mediated by a PGS for cognitive‐function. Future directions include assessing the contribution of PGS to cognition over time, and investigating how environmental and life‐course factors may moderate the association between PGS and cognition.

